# The clinical and radiographic features of eastern equine encephalitis: A single-center retrospective case series

**DOI:** 10.1097/MD.0000000000041170

**Published:** 2024-12-27

**Authors:** Maria A. Garcia-Dominguez, Majaz Moonis, Vincent Kipkorir, Bahadar S. Srichawla

**Affiliations:** a Department of Neurology, University of Massachusetts Chan Medical School, Worcester, MA; b Department of Medicine, University of Nairobi, Nairobi, Kenya.

**Keywords:** arbovirus, eastern equine encephalitis, eastern neuroinfectious diseases, equine encephalitis virus, flavivirus, neuroimmunology

## Abstract

**Rationale:**

This case series aims to describe the clinical and radiographic findings associated with eastern equine encephalitis (EEE) virus.

**Patient concerns:**

Patients in this series presented with a variety of neurological symptoms, including altered mental status, seizures, and focal neurological deficits. Common initial concerns included confusion, hemiparesis, fever, and flu-like symptoms. In all cases, the progression of neurological symptoms prompted urgent medical evaluation and hospitalization.

**Diagnoses:**

Diagnosis of EEE was confirmed in all 4 patients by detection of EEE-specific IgM antibodies in cerebrospinal fluid. Cerebrospinal fluid studies also showed elevated protein, red blood cells, normal or high glucose, and elevated white blood cells with lymphocytic or neutrophil predominance. Magnetic resonance imaging showed T_2_ hyperintensities in the basal ganglia, thalamus, and cortical regions. Two cases showed encapsulation as evidence by a hyperintense ring around the basal ganglia or thalamus. Electroencephalogram findings ranged from normal, focal irritability, and spikes to nonconvulsive status epilepticus. Clinical and/or electrographic evidence of seizure was seen in all 4 cases.

**Interventions:**

All patients received supportive care, including anticonvulsant therapy, often using levetiracetam. Two patients were treated with high-dose intravenous methylprednisolone and one with intravenous immunoglobulin (IVIG) to modulate the immune response. Empiric antibiotics and antivirals were initiated in most cases until the diagnosis of EEE was confirmed. Two patients required intubation and mechanical ventilation, one of which was due to seizure activity and nonconvulsive status epilepticus.

**Outcomes:**

We report a 1/4 (25%) mortality rate. The average hospital stay among survivors was 10 days. Two-fourth (50%) required intubation and mechanical ventilation. Two patients were discharged to rehabilitation facilities, one patient recovered fully with resolution of magnetic resonance imaging abnormalities at follow-up, and one patient experienced a fatal outcome after the family opted to withdraw care due to a poor prognosis.

**Lessons:**

This case series underscores the importance of maintaining a high clinical suspicion of EEE in patients with acute neurological symptoms, especially in endemic areas. Early diagnosis and treatment are crucial, but the variability in presentation and imaging findings complicates this process. Early use of high-dose steroids can improve outcomes.

## 1. Introduction

Arboviruses are a continuing and reemerging threat to global health.^[1]^ Eastern equine encephalitis (EEE) is a rare but severe arboviral infection transmitted primarily by the bite of infected mosquitoes. First identified in the 1930s in North America, EEE has since been recognized as one of the most lethal mosquito-borne diseases in the United States, with a fatality rate of cases approaching 30% to 50% in symptomatic individuals.^[2]^ Survivors of EEE often experience significant neurological sequelae, making this disease a critical concern for both public health and clinical practice.^[3]^

The etiological agent of EEE is the eastern equine encephalitis virus (EEEV), a member of the *Alphavirus* genus within the *Togaviridae* family. The virus predominantly circulates in bird populations within freshwater swamps, with occasional spillover to horses and humans.^[4]^ Although human cases remain relatively rare, the severity of the disease, coupled with its potential for outbreaks, requires a greater awareness and understanding of its clinical manifestations, diagnostic challenges, and management strategies.^[5]^

In recent years, climate fluctuations and changes in land use have been associated with alterations in the geographic distribution and incidence of EEE, raising concerns about the potential for increased human exposure.^[6]^ Despite advances in diagnostic techniques and supportive care, the treatment of EEE remains primarily symptomatic, with no specific antiviral therapies available.^[7]^ The high morbidity and mortality associated with EEE underscores the need for continued research and surveillance to better characterize the disease and improve patient outcomes.^[8]^

This retrospective case series aims to provide a comprehensive review of EEE cases, focusing on clinical presentation, diagnostic approaches, management strategies, and results. By analyzing these cases, we seek to contribute to the growing body of knowledge about EEE and offer insights that can inform future clinical practice and public health interventions. This case series was completed in accordance with the established CARE guidelines for case reports/series.^[9]^

## 2. Methods

### 2.1. Study design and setting

This study is a retrospective case series from Massachusetts focusing on patients diagnosed with EEE between January 2019 and August 2024. Informed consent was obtained from all patients or their legal guardians prior to inclusion in the study.

### 2.2. Case identification and data collection

Cases were identified through a comprehensive search of the electronic medical records system at the University of Massachusetts Chan Medical School. The inclusion criteria were patients with a confirmed diagnosis of EEE based on the presence of EEE IgM antibodies. The exclusion criteria included patients with incomplete medical records or those who did not meet the diagnostic criteria for EEE. Data were extracted from medical records, including demographic information (age, sex, and race/ethnicity), clinical presentation (symptoms, duration of the illness), laboratory findings (cerebrospinal fluid [CSF] analysis, serology, and polymerase chain reaction results), imaging studies (magnetic resonance imaging [MRI], computerized tomography [CT]), treatment received (antiviral therapy, supportive care), and outcomes (mortality, neurological sequelae, length of hospital stay). The data collection process was standardized by the researchers to ensure accuracy and consistency.

### 2.3. Statistical analysis

Descriptive statistics were used to summarize the demographic and clinical characteristics of the patients. Continuous variables were reported as means with standard deviations or medians with interquartile ranges, depending on the distribution of the data. Categorical variables were presented as frequencies and percentages. All statistical analyses were performed using R-Studio (2024.04.2+764).

### 2.4. Ethical considerations

This study was conducted in accordance with the ethical principles outlined in the Declaration of Helsinki. Informed consent was obtained from all participants or their legal guardians prior to inclusion in the study. Patient confidentiality was maintained throughout the study, with all data being deidentified and securely stored. The datasets generated during and/or analyzed during the current study are available from the corresponding author on reasonable request.

## 3. Results

### 3.1. Case 1

**Figure 1.**
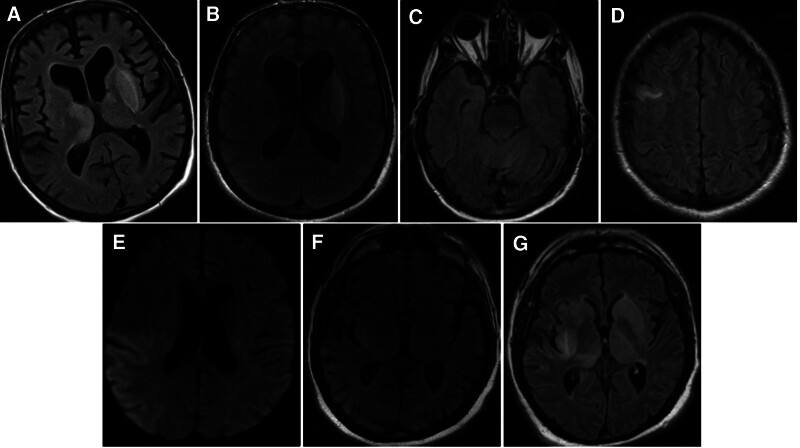
(A) Case 1: increased T_2_ weighted signal in FLAIR sequence within the left basal ganglia and medial aspect of the right thalamus. (B) Case 1: interval improvement without contrast enhancement. (C) Case 2: increased T_2_ signal on the FLAIR sequence involving the right mesial temporal lobe. (D) Case 3: linear focus of T_2_ hyperintense signal abnormality within the subcortical white matter of a single gyrus in the right frontal lobe, suggestive of cerebritis. (E) Case 4: acute infarct in the territory of the right posterior cerebral artery, specifically involving the right thalamic pulvinar. (F) Case 4: large amorphous area of faint T_2_ hyperintensity with diffusion restriction affecting the right temporal-parietal gyri, insula, sub-adjacent white matter, and basal ganglia. (G) Case 4: increased T_2_ signal in the basal ganglia and thalamic regions bilaterally, with the left side more affected than the right. FLAIR = fluid-attenuated inversion recovery.

An 85-year-old male with a medical history of benign prostatic hyperplasia presented to the emergency department with altered mental status and a generalized tonic-clonic seizure. His condition rapidly deteriorated, resulting in a decreased level of consciousness, which required endotracheal intubation for protection of the airway. Initial CT and computerized tomography angiography (CTA) of the head revealed no acute intracranial abnormalities but identified chronic occlusion of the left internal carotid artery and an ulcerative plaque in the right internal carotid artery. Subsequent MRI of the brain without contrast showed an increased T_2_ signal in fluid-attenuated inversion recovery images within the left basal ganglia and medial aspect of the right thalamus (Fig. 1A and B). A 72-hour continuous electroencephalogram (EEG) demonstrated frequent generalized periodic discharges with frontally maximal triphasic morphology at a frequency of 1.5 to 2.0 Hz. Lumbar puncture revealed CSF analysis with elevated protein levels (101 mg/dL), 29 white blood cells (WBCs)/mm³ with 79% monocytes/macrophages. An infectious meningitis/encephalitis panel detected positive EEE IgM antibody via enzyme immunoassay (EIA), confirming the diagnosis. The patient was treated with high-dose intravenous methylprednisolone (1000 mg daily for 5 days). Anticonvulsant therapy with levetiracetam (1250 mg twice daily), and valproate (750 mg twice daily) was started. The patient had a significant risk factor for exposure as an avid gardener, spending several hours outdoors daily. A repeat MRI on hospital day 7 showed an improvement in the interval with no contrast enhancement. The patient was successfully extubated on hospital day 6 and subsequently discharged to an acute rehabilitation facility on hospital day 11 for further recovery and rehabilitation.

### 3.2. Case 2

A 77-year-old man with a history of aortic valve replacement on chronic warfarin therapy presented to the emergency department with several transient episodes of altered mental status. The patient experienced 5 to 6 episodes of confusion, each lasting several hours to days, mainly affecting his ability to recall history and causing disorientation to time and place. There was no impairment in activities of daily living. The initial CT and CTA of the head were unremarkable. However, MRI of the brain with and without contrast revealed increased T_2_ signal on the fluid-attenuated inversion recovery sequence involving the right mesial temporal lobe, raising concerns for a possible seizure episode (Fig. 1C). Susceptibility-weighted imaging showed scattered punctate foci of microbleeds at the gray-white matter junction, which were considered chronic.

A continuous EEG recorded intermittent sharp waves in the right frontal area during sleep, further supporting seizure-related etiology. CSF analysis revealed 21 WBCs/mm³ with 52% neutrophil predominance, normal glucose levels, and elevated protein (55 mg/dL). The presence of 8700 red blood cells (RBCs)/mm³ in tube 1 and 17,250 RBCs/mm³ in tube 4 was noted. An infectious panel, including meningitis, autoimmune, and viral encephalitis tests, identified an elevated EEE IgM in the CSF via EIA, confirming the diagnosis of EEE. The patient was started on anticonvulsant therapy with levetiracetam (750 mg twice daily) and closely monitored. Despite the severe nature of the disease, the patient remained stable and was discharged to a rehabilitation facility on hospital day 12 for further recovery and continued treatment of his neurological symptoms. A repeat MRI of the brain without contrast was completed in 6 months that showed resolution of previous mesial temporal sclerosis and the patient continued to remain seizure free.

### 3.3. Case 3

A 29-year-old male came to the emergency department following a generalized tonic clonic seizure. His symptoms began with flu-like manifestations, including abdominal pain, diarrhea, muscle cramps, nausea, and a fever of 101 °F. The sudden onset of seizure activity prompted further investigation. CSF analysis revealed an elevated protein level of 146 mg/dL, normal glucose of 60 mg/dL, 28 RBCs/mm³, and 310 white blood cells WBCs/mm³, with a differential showing 77% neutrophils and 17% lymphocytes. Given the initial findings, the patient was empirically treated for bacterial meningitis and herpes simplex virus encephalitis with acyclovir, ceftriaxone, and vancomycin. MRI of the brain demonstrated a linear focus of T_2_ hyperintense signal abnormality within the subcortical white matter of a single gyrus in the right frontal lobe, suggestive of cerebritis (Fig. 1D). A comprehensive meningitis/encephalitis panel was performed in the CSF, which confirmed the presence of EEE IgM via EIA. A routine EEG was conducted, which returned normal results. The patient started anticonvulsant therapy with levetiracetam (750 mg twice daily). His condition stabilized and he was discharged from the hospital on day 7. A follow-up MRI of the brain, completed 3 years later, showed complete resolution of the previously identified lesion, indicating full recovery from the initial encephalitic process.

### 3.4. Case 4

A 70-year-old man with a medical history of hypertension, type 2 diabetes mellitus, coronary artery disease status after percutaneous coronary intervention, and hyperlipidemia presented to the emergency department with acute left-sided hemiparesis of acute onset and twitching of the left upper extremity. He was activated as a code stroke with a National Institutes of Health Stroke Scale score of 13, primarily due to dense left-sided hemiplegia and sensory deficits. Initial head CT and CTA did not show acute abnormalities or large vessel occlusion. The patient was loaded with 2000 mg of levetiracetam. Subsequently, he developed a high fever of 39.7 °C. Given the clinical picture, empiric antibiotics, and antivirals were started for suspected meningitis/encephalitis, including ceftriaxone, vancomycin, and acyclovir. MRI of the brain revealed an acute infarct in the right posterior cerebral artery territory, specifically involving the right thalamic pulvinar. Additionally, there was a larger amorphous area of faint T_2_ hyperintensity with diffusion restriction involving the right temporal-parietal gyri, insula, sub-adjacent white matter, and basal ganglia (Fig. 1E and F). CSF analysis showed elevated glucose (82 mg/dL), protein (140 mg/dL), 11 RBCs/mm³, and 8 WBCs/mm³, with a differential of 44% lymphocytes, 27% neutrophils, and 29% monocytes/macrophages. A meningitis/encephalitis panel returned positive for EEE IgM in the CSF via EIA. The patient’s hospital course was complicated by generalized tonic-clonic seizures, which required intubation and mechanical ventilation. He was loaded with fosphenytoin and started on a maintenance dose. Continuous EEG monitoring revealed evidence of nonconvulsive status epilepticus originating in the right hemisphere. On hospital day 5, a repeat MRI of the brain showed evolution of the previously identified acute infarction and increased T_2_ signal in the basal ganglia and thalamic regions bilaterally, with the left side more affected than the right (Fig. 1G). Given the worsening clinical picture, the patient started on high-dose intravenous methylprednisolone (1000 mg daily) and intravenous immunoglobulin (IVIG) on hospital day 7. Unfortunately, on day 8 of hospitalization, the patient developed ventilator-associated pneumonia. Due to the poor prognosis and the patient’s declining condition, the family opted to withdraw care. A full summary of the clinical and radiographic findings of call reported cases are provided in Table 1.

**Table 1:** Summary of clinical characteristics, diagnostic findings, management, and results in 4 patients with EEE.

Age	Gender	Clinical symptoms	MRI findings	EEG findings	CSF studies	Management	Length of hospital stay	Outcomes
85	Male	Altered mental status, decreased consciousness	Increased T2 signal in left basal ganglia, right thalamus	Frequent frontally maximal GPDs with triphasic morphology	Protein 101 mg/dL, WBC 29/mm^3^, 79% monocytes/macrophages, Positive EEE IgM	Intubation, IV methylprednisolone, IVIG, Levetiracetam	11	Discharged to acute rehabilitation
77	Male	Transient altered mental status, confusion	Increased T2 signal in right mesial temporal lobe, microbleeds	Intermittent right frontal sharp waves during sleep	Protein 55 mg/dL, WBC 21/mm³, 52% neutrophils, Positive EEE IgM	Levetiracetam, Empiric antibiotics and antivirals	12	Discharged to rehabilitation facility
29	Male	Flu-like symptoms, seizure	T2 hyperintense signal in right frontal lobe	Normal EEG	Protein 146 mg/dL, WBC 310/mm³, 77% neutrophils, Positive EEE IgM	Levetiracetam, Empiric antibiotics and antivirals	7	Discharged from hospital, resolved on follow-up MRI
70	Male	Left-sided hemiparesis, seizure	Acute infarct in right PCA territory, T2 hyperintensity in right temporal-parietal regions	Nonconvulsive status epilepticus from right hemisphere	Protein 140 mg/dL, WBC 8/mm³, 44% lymphocytes, Positive EEE IgM	Intubation, Fosphenytoin, IV methylprednisolone, IVIG, Empiric antibiotics and antivirals	8	Care withdrawn due to poor prognosis

CSF = cerebrospinal fluid, GPDs = generalized periodic discharges, MRI = magnetic resonance imaging.

## 4. Discussion

**Figure 2.**
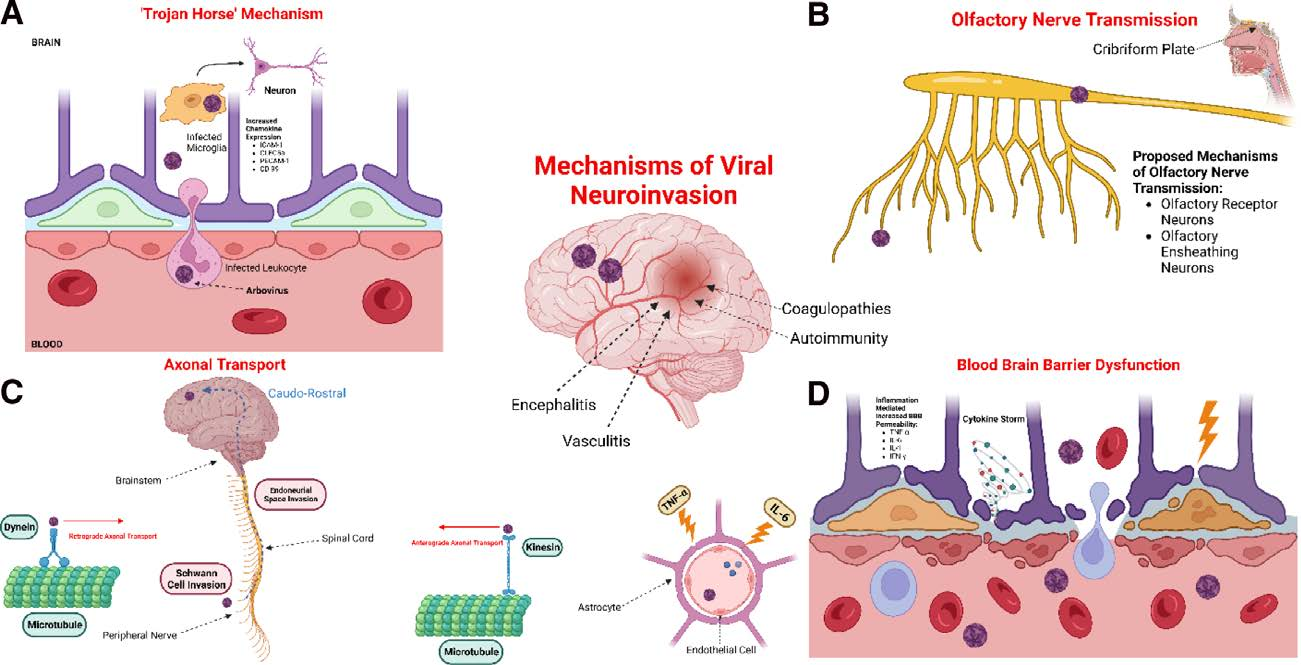
Mechanisms of viral neuroinvasion including entry via (A) the “trojan horse” mechanism, (B) olfactory nerve transmission, (C) axonal transport, and (D) blood-brain barrier dysfunction (adapted from the corresponding author Srichawla et al^[10]^).

EEE is a rare but serious arboviral disease with a high mortality rate and significant long-term neurological sequelae among survivors.^[2]^ This case series highlights the diverse clinical manifestations, diagnostic challenges, and treatment outcomes of EEE in 4 patients, underscoring the importance of early recognition and aggressive management. The clinical presentation of EEE can be varied, ranging from nonspecific flu-like symptoms to severe neurological deficits, as demonstrated in our cases. The EEE virus can cause neuroinvasive disease through various mechanisms, including the trojan horse mechanism and inflammation of the blood–brain barrier (Fig. 2).^[10]^ Neuroinvasive disease and subcortical lesions have been reported in other arboviral infections such as the West Nile virus.^[11]^ Common symptoms in this series included altered mental status, seizures, and focal neurological deficits. Variability in presentation, particularly in the elderly population, often complicates the initial diagnosis, leading to delays in appropriate treatment. The neuroimaging findings were heterogeneous, with MRI often showing T_2_ hyperintensities in areas such as the basal ganglia, thalamus, and cortical regions. These findings, although not pathognomonic, can raise suspicion for encephalitic processes, particularly when combined with clinical and CSF findings. The presence of microbleeds in some cases, as seen in Case 2, may further complicate the differential diagnosis. Serious consideration must be given to empiric treatment with high-dose steroids and IVIG in patients with clinical sequelae consistent with infection and radiographic evidence of subcortical encapsulation. The diagnosis of EEE was confirmed in all cases by detecting EEE-specific IgM antibodies in the CSF, a critical step that distinguishes EEE from other causes of viral encephalitis. However, this diagnostic approach is limited by the availability of testing and the potential for cross-reactivity with other arboviruses. The management of EEE is primarily supportive, with no specific antiviral therapy available.^[8]^ Although previous retrospective studies have shown that early high-dose steroids and IVIG may be beneficial in reducing morbidity and mortality.^[12]^ In some cases, patients in this series received anticonvulsants, high-dose corticosteroids, and IVIG. Despite these interventions, the results were mixed, reflecting the aggressive nature of the disease. One patient in our series succumbed to complications despite intensive care, highlighting the high mortality associated with EEE, particularly in older adults with comorbidities. The use of high-dose corticosteroids as seen in case 1, may offer some benefit in reducing inflammation and modulating the immune response.

### 4.1. Limitations and future directions

The use of high-dose corticosteroids and IVIG, as seen in Cases 1 and 4, may offer some benefit in reducing inflammation and modulating the immune response, although the evidence supporting these treatments is limited and mainly anecdotal. More research is needed to establish standardized treatment protocols. Major limitations of this case series include its retrospective nature, lack of a control group, observer bias, small sample size and limited generalizability. Future research should focus on developing more effective antiviral therapies and immunomodulatory treatments for EEE. In addition, more robust diagnostic tools are needed that can provide rapid and accurate detection of EEE, potentially reducing diagnosis time and improving patient outcomes. Public health efforts should also focus on preventive measures, such as vector control and vaccine development, to reduce the incidence of this devastating disease.^[1]^

## 5. Conclusions

EEE is a rare but devastating neuroinvasive disease with high morbidity and mortality, particularly in older adults and individuals with underlying comorbidities. This case series highlights the wide spectrum of clinical presentations, from flu-like symptoms to severe neurological impairment, underscoring the importance of maintaining a high index of suspicion for EEE in endemic areas. The diagnosis of EEE is highly dependent on the detection of EEE-specific IgM antibodies in the cerebrospinal fluid, which, when combined with neuroimaging findings and clinical presentation, can confirm the diagnosis. The CSF studies showed elevated protein, WBCs with lymphocytic or neutrophilic predominance, elevated RBCs and normal or high glucose. Clinical and/or electrographic evidence of seizures was seen in all patients. And MRI often showed encapsulation of subcortical structures, focal cerebritis, and cortical diffusion restriction. Serious consideration should be given for empiric treatment with high-dose steroids and IVIG in patients with the clinical sequelae consistent with an infection and radiographic evidence of encapsulation of subcortical structures. The mixed outcomes observed in this case series emphasize the aggressive nature of the disease and the need for early intervention and supportive care. The use of immunomodulatory therapies, such as high-dose corticosteroids and IVIG, can offer potential benefits, but its efficacy has not yet been validated in larger studies.

## Author contributions

**Conceptualization:** Maria A. Garcia-Dominguez, Bahadar S. Srichawla.

**Data curation:** Vincent Kipkorir, Bahadar S. Srichawla.

**Formal analysis:** Bahadar S. Srichawla.

**Funding acquisition:** Bahadar S. Srichawla.

**Investigation:** Bahadar S. Srichawla.

**Methodology:** Maria A. Garcia-Dominguez, Bahadar S. Srichawla.

**Project administration:** Bahadar S. Srichawla.

**Resources:** Bahadar S. Srichawla.

**Software:** Bahadar S. Srichawla.

**Supervision:** Bahadar S. Srichawla.

**Validation:** Bahadar S. Srichawla.

**Visualization:** Bahadar S. Srichawla.

**Writing – original draft:** Maria A. Garcia-Dominguez, Bahadar S. Srichawla.

**Writing – review & editing:** Maria A. Garcia-Dominguez, Majaz Moonis, Bahadar S. Srichawla.
